# Chemopreventive Efficacy of Thymoquinone in Chemically Induced Urinary Bladder Carcinogenesis in Rat

**DOI:** 10.1155/2022/6276768

**Published:** 2022-09-15

**Authors:** Karmand Salih Hamaamin, Bushra Hassan Marouf

**Affiliations:** Department of Pharmacology and Toxicology, College of Pharmacy, University of Sulaimani, Sulaymaniyah, 46001 Kurdistan Region, Iraq

## Abstract

The effects of thymoquinone (TQ) in a carcinogen-based models of urinary bladder cancer were evaluated, using 45 male rats in five groups. In negative control (*n* = 10), only tap water was given. In positive control (*n* = 10), the rats received 0.05% N-butyl-N-(4-hydroxybutyl)-nitrosamine (BBN) in drinking water for 9 weeks. In preventive groups with 25 mg/kg (*n* = 10) and 50 mg/kg (*n* = 10), oral TQ was concurrently given with 0.05% BBN for 9 weeks and continued for one more week after cessation of BBN. Preventive-treatment group (*n* = 5) received 50 mg/kg TQ orally for 20 weeks. Five rats from each group were sequentially sacrificed in two phases: the induction phase at 12th week (except the last group) and the rest in postinduction phase at 20th week. The bladders were examined macroscopically for lesion formation, and the masses were submitted for histopathological evaluation. Markers for total oxidant status (TOS), inflammation (nuclear factor kappa B (NF-*κ*B)), and angiogenesis (vascular endothelial growth factor (VEGF)) were also assessed. There was a reduced number of bladder lesions in the TQ groups versus the carcinogen group at both phases. Histopathological findings demonstrated a significant improvement in the abnormal morphological changes in the urothelium of the TQ-treated groups. Thymoquinone exerted a significant antioxidant and anti-inflammatory effect by a decrease in serum level of TOS and NF-*κ*B at week 12 which was maintained low in phase two at week 20. The serum level of VEGF was also alleviated in the induction phase at week 12 and maintained low in postinduction period. In TQ preventive-treatment approach, a nonsignificant elevation of serum level of TOS and NF-*κ*B and slight reduction in VEGF were observed at the end of the experiment. These data suggest that TQ may be effective in preventing bladder carcinogenesis, and the suggested mechanisms might be related to antioxidant, prooxidant, and anti-inflammatory properties of TQ.

## 1. Introduction

Urinary bladder cancer is one of the most common malignant diseases of the urinary tract system. It is the ninth most common cancer worldwide and one of the leading causes of cancer-associated deaths in developed countries [1]. Despite the advanced progress in the conventional cancer therapeutic approaches, their clinical applications are still not convincing due to the serious adverse effects that accompany the use of these medications, multidrug resistance, and potential recurrence [2]. Hence, with poor response to therapies, combined with drug resistance, the dissatisfaction of cancer patients with this conventional therapy and subsequent chemotherapeutic adverse effects necessitate the development and establishment of novel treatment protocols. All these factors have led to the expansion of the field of antitumor treatment toward natural herbal extracts as an alternative therapy. Therefore, a large amount of research has studied the natural plant products and their bioactive compounds in an attempt to find new and better treatments to bladder tumors.

Thymoquinone (TQ) “2-isopropyl-5-methyl-1, 4-benzoquinone” is the main active constituent of the *Nigella sativa Linn* (black cumin) seed oil extract which has been traditionally used in the Middle East and Southeast Asian countries as the blessing seed. It exhibits pleiotropic activity including anti-inflammatory, antihistaminic, antihypertensive, hypoglycemic, immune enhancement, and antiarthritic [3, 4]. Furthermore, it has anticancer and antioxidant actions in various types of cancers such as breast, bone, cervix, liver, colon, blood, oral, head and neck, prostate, kidney, and bladder [5–7].

In the last decade, many preclinical studies have evaluated that the potential cytotoxic effect of TQ and its anticancer actions has been elucidated by dampening the initiation, proliferation, invasion, migration, and progression of cancer [8].

Thymoquinone exhibits anticancer activity via numerous mechanisms, including antioxidant that interferes with DNA structure, and ability to regulate many genetic pathways and alter carcinogenic signaling molecules/pathways and immunomodulation; it induces apoptosis in various cancer cell types through many cellular targets such as phosphorylation of nuclear factor *κ*B (NF-*κ*B) and decreasing the extracellular signal–regulated kinases 1/2 (ERK1/2) and phosphatidylinositol-4,5-bisphosphate 3-kinase (PI3K) activities [9].

Additionally, anticancerous effect of TQ mainly occurs by modulating different cell signaling pathways such as Bcl2/Bax ratio, vascular endothelial growth factor (VEGF), p53, NF-*κ*B, and other oncogenes [10, 11] as depicted in breast cancer models that TQ can attenuate VEGF, enhance serum INF-*γ* levels, and suppress angiogenesis [12].

Despite these advances in targeting TQ as a treatment approach for cancer, no study has elucidated the preventive strategy of TQ in urothelial cancer in preclinical study. For this reason, the aim of designing the present study was to assess the potential chemopreventive effects of TQ in a chemically induced bladder cancer model (urothelial cancer) using organ-specific carcinogen N-butyl-N-(4-hydroxybutyl) nitrosamine (BBN) in albino rats.

## 2. Material and Methods

### 2.1. Reagents

All the reagents and laboratory kits used in the present study were of high purity and analytical grade. Thymoquinone (TQ) was from Glentham Life Sciences (United Kingdom); N-butyl-N-(4-hydroxybutyl) nitrosamine (BBN) was from Tokyo Chemical Industry Co., Ltd. (Tokyo, Japan). Rat total oxidant status (TOS) ELISA kit, nuclear factor kappa B (NF-*κ*B) ELISA kit, and vascular endothelial growth factor (VEGF) ELISA kit were from Bioassay Technology Laboratory (Harborne Road, Birmingham, England).

### 2.2. Animals

Wistar albino male rats of 6-8 weeks (weighing 160 ± 20 g) were used in the study; they were obtained from the animal house of University of Sulaimani. The rats were housed in plastic cages and acclimatized to the standardized environment (12 h light–dark cycle, a temperature of 25 ± 1°C, and a relative humidity of 50 to 70%) for 1 week with standard animal diet and free access to water *ad libitum*.

### 2.3. Induction of Bladder Carcinogenesis

The bladder cancer was induced by the organ-specific carcinogen N-butyl-N-(4-hydroxybutyl) nitrosamine (BBN) in a dose of 0.05% in drinking water for 9 weeks. This method is a highly followed way for bladder tumor induction in rodents [13]. The drinking water was changed every day, and the bottles were covered with aluminum foil to prevent light exposure. This model has been extensively used to evaluate agents that might prevent urinary bladder cancer [14]. All procedures of the work were conducted in accordance with the Declaration of Helsinki on the welfare of experimental animals and with the approval of the Ethical Committee of College of Pharmacy, University of Sulaimani, under registration number PH36-21 in November 14, 2021.

### 2.4. Animal Groups and Treatment Protocol

**Figure 1:**
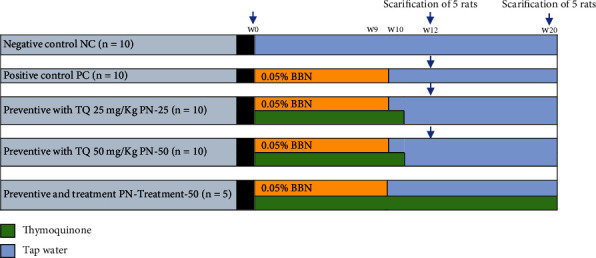
Schematic representation of the study protocol. TQ: thymoquinone; BBN: N-butyl-N-(4-hydroxybutyl) nitrosamine; W: week; *n*: number of rats.

The schematic representation of the experiment protocol is demonstrated in Figure 1. Forty-five rats were randomly divided into five group. In negative control (NC) (*n* = 10), animals received only tap water across the study. In positive control (PC) (*n* = 10), animals were given 0.05% BBN in drinking water for 9 weeks for tumor induction. Preventive groups with low-dose TQ (*n* = 10) and preventive group with high-dose TQ (*n* = 10), here 25 mg/kg and 50 mg/kg oral TQ, respectively, were concurrently given with 0.05% BBN for 9 weeks and continued for one more week after cessation of BBN. Last group was the preventive-treatment group (PN-Treatment 50) (*n* = 5); this group received TQ 50 mg/kg starting from day one concurrently with 0.05% BBN in drinking water for 9 weeks and continued till week 20. The experimental design consisted of two phases. Phase one was a period of tumor induction from day one to week 12, while phase two was postinduction period; it starts from week 12 till week 20.

At the end of 12th week, five rats from each NC, PC, PN-25, and PN-50 groups were anesthetized and euthanized with humane practice to investigate the effect of preventive approach of TQ on macroscopical, microscopical, and biochemical parameters in phase one. While at the end of 20^th^ week, the remaining five rats in the mentioned groups plus all rats in the PN-Treatment 50 group were sacrificed to investigate the long-lasting preventive approach of TQ in phase two. The protocol of this study was obtained from a previous work with some modification [15], and the dose of TQ was selected based on the previous studies [9, 16].

Before removal of the bladder from each animal, it was filled intravesically through catheterization with 10% formaldehyde as prefixation for histological analysis; then, a ligature was placed around the bladder neck to maintain proper distention then immediately removed; after 24 hours, the bladder was opened anteroposteriorly and photographed.

During the experimental period, body weight and visual physical signs and volume of drinking water were monitored and recorded twice weekly.

### 2.5. Gross Pathology (Macroscopic Analysis)

Prefixed bladders with 10% formaldehyde of all animals of phase one (week 12) and phase two (week 20) were incised; then, bladder's lumen was checked for grossly visible lesions, and the number of lesions per each rat was recorded. Furthermore, the bladder was analyzed macroscopically for urothelium texture and thickness.

### 2.6. Histological Quantitative Evaluation of Urothelium (Microscopic Analysis)

#### 2.6.1. Histotechnique Preparation

Following macroscopic assessment, bladder samples were cross sectionally cut and immobilized into tissue cassettes then fixed with 10% buffered formaldehyde solution. Afterward, the tissue samples were processed and dehydrated through series of ascending concentrations of ethanol alcohol (50%, 60%, 70%, 80%, 90%, and 100%), followed by couple steps of xylene clearance. Next, the processed samples were infiltrated and embedded in melted paraffin blocks. With the aid of semiautomated rotary microtome, paraffinized tissues were sectioned to 5 *μ*m. Then after, tissue sections were fixed on glass slides dried on hot plate then deparaffinized and cleaned with two steps xylene changes, later on placed in a hot oven at 50°C for 30 minutes. Finally, bladder sections were stained with Harris' hematoxylin and eosin solution, cleaned with xylene and cover slipped, and then examined and evaluated quantitatively under light microscope.

#### 2.6.2. Microscopic Quantitative Lesion Grading of the Urothelium

In general, urothelial lesion scoring-grading system was evaluated quantitatively using the ImageJ analyzer software (AmScope, 3.7) via a uniocular lens camera (MD500, 2019) fixed with bright field light microscope (NOVEL XSZ-N107T, China). Urothelial preneoplastic (hyperplasia and dysplasia) transformation was estimated and measured in percentage of calculated cell numbers from randomly selected different fields within a bladder section, whereas papillary-like growth was assessed in *μ*m and statistically evaluated as mean percentage. While the areas of neoplastic transformation (carcinoma in situ (Cis)) and infiltrative growth were evaluated in *μ*m from randomly chosen urothelium fields under higher magnification lens (100x), then the mean average was considered statistical in percentage. Lastly, the mean percentage of all calculated values was expressed as the following grading system: score 0-10% as normal with no atypia, score 10-25% as low grade means presence of atypia, score 25-50% as medium grade means dysplasia, score 50-75% as high grade refers to carcinoma in situ (Cis), and score 75-100% as critical grade means infiltrative or invasive neoplastic transformation.

### 2.7. Serum Biochemical Analysis

The blood samples were immediately collected by cardiac puncture in tubes with no anticoagulant. Serum was separated by centrifugation at 5000 rpm for 10 minutes and then stored at -70°C for analysis of serum levels of total oxidant status (TOS), nuclear factor kappa B (NF-*κ*B), and vascular endothelial growth factor (VEGF).

### 2.8. Statistical Analysis

Analyses was performed using the GraphPad Prism software, LCC, version 9.3. Continuous quantitative variables were analyzed with the ordinary one-way analysis of variance (ANOVA) test followed by Tukey's test. Data with different time point such as body weight was analyzed with two-way ANOVA repeated measure multiple comparison followed by Tukey's test to show the statistical differences between the groups with different time points. In all cases, the level of statistical significance was set at *P* < 0.05.

## 3. Results

### 3.1. Survival Rate and Body Weight

**Figure 2:**
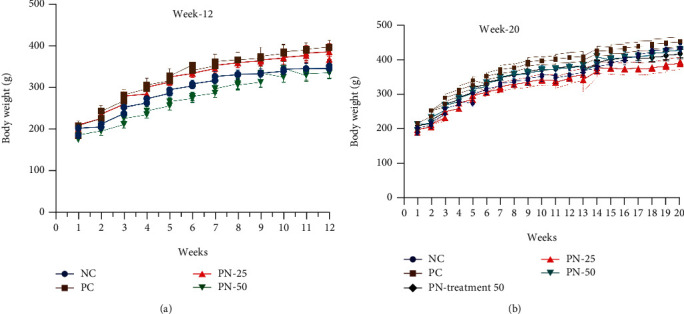
The body weight difference between the studied groups in BBN-induced model of bladder cancer at (a) week 12 and (b) week 20. Data are presented as mean ± SEM. NC: negative control group; PC: positive control group; PN-25: preventive group with 25 mg/kg TQ; PN-50: preventive group with 50 mg/kg TQ; PN-Treatment 50: prevention-treatment group. One-way ANOVA repeated measure multiple comparison test was used followed by Tukey's multiple comparison to determine the differences between the groups. There were no significant differences in weight gain during the whole experimental period (*P* > 0.05).

All the rats survived and completed the entire study period; there were no noticeable changes in food and water consumption. Furthermore, no changes detected in physical behavior and appearance among the rats of the all groups. Body weight was measured every three days (i.e., twice weekly), and there were no significant changes in weight gain in different time points between the TQ-treated groups compared to the positive controls during the entire experiment (Figure 2).

### 3.2. Macroscopic Description of the Bladder Urothelium

**Figure 3:**
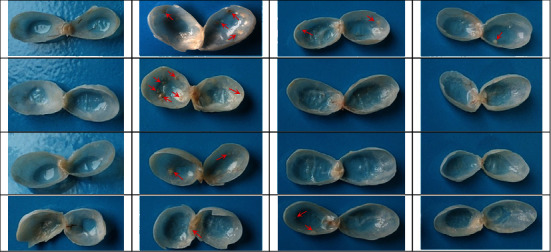
Macroscopic presentation of the bladder urothelium of the representative animals at week 12. All bladders of the negative control (NC) group reveal a normal appearance. Animals from the positive control (PC) group show a higher number of lesions (shown by the arrows) in comparison to the PN-25 and PN-50 groups, in which their animals were given 25 mg/kg and 50 mg/kg thymoquinone (TQ), respectively.

**Figure 4:**
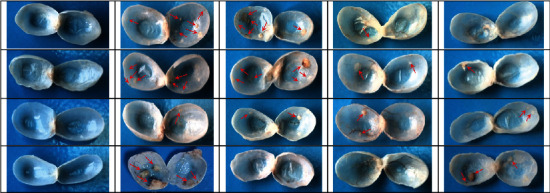
Macroscopic presentation of the bladder urothelium of the representative animals at the end of week 20. All bladders of the negative control (NC) group reveal a normal appearance. Animals from the positive control (PC) group show higher number of aggressive lesions (shown by the arrows) in comparison to the PN-25 and PN-50 groups, in which their animals were given 25 mg/kg and 50 mg/kg thymoquinone (TQ), respectively. Preventive-treatment (PN-Treatment 50) protocol shows lesser tumor lesions with less multiplicity.

**Figure 5:**
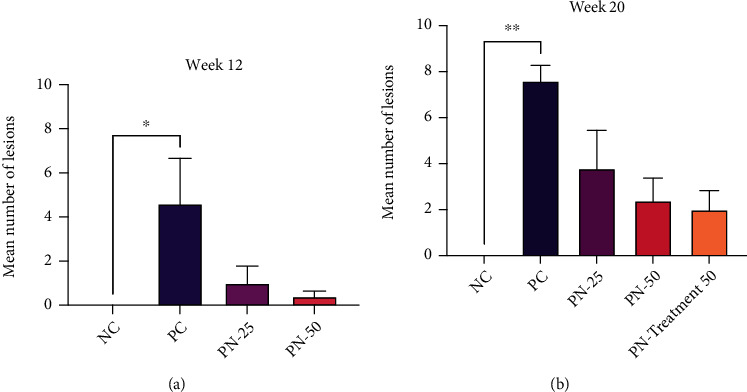
Number of urothelial lesions in each group at (a) week 12 and (b) week 20. Values are expressed as mean ± SEM (standard error of mean); Kruskal-Wallis test was used to determine the differences between the groups. ^∗^*P* value < 0.03 and ^∗∗^*P* value < 0.002 are statistically significant.

Macroscopic presentation of bladder urothelium in all groups at weeks 12 and 20 is shown in Figures 3 and 4. Initially at week 12, the bladders from positive control group (PC) showed multiple visible lesions, while use of TQ in the preventive groups (PN-25 and PN-50) caused marked inhibition of bladder lesion formation. The number of lesions in each group was presented as mean ± SEM; in the PC group, it was 4.6 ± 2.06; meanwhile, the concomitant use of 25 mg/kg and 50 mg/kg TQ with BBN as a preventive approach in the PN-25 and PN-50 groups, respectively, resulted in a noticeable suppression of the tumor induction and decreasing number of lesions to 1.73 ± 0.77 and 0.54 ± 0.24 in the PN-25 and PN-50 groups, respectively (Figure 5(a)).

In phase two and precisely at week 20 of the experiment, the bladder's urothelium was examined for tumor growth in both preventive approaches, in which TQ was given from day one to week 10, and in preventive and treatment approach, in which 50 mg/kg of TQ in group PN-Treatment 50 was given for 20 weeks from first day until the end of the experiment. The number of lesions in each group was also expressed as mean ± SEM. All the rats in PC had easily detected lesions by naked eye which was 7.6 ± 0.67; however, TQ showed long-lasting preventive effect in postinduction period by reduction in the number of lesions of both groups to 3.8 ± 1.65 and 2.4 ± 0.97, respectively, compared with the PC group. Additionally, preventive and treatment protocol resulted in a better treatment outcome as evidenced by a decrease in average number of lesions per each rat to 2 ± 0.83 compared to the BBN group (Figure 5(b)).

### 3.3. Microscopically Quantitative Histopathological Analysis

**Figure 6:**
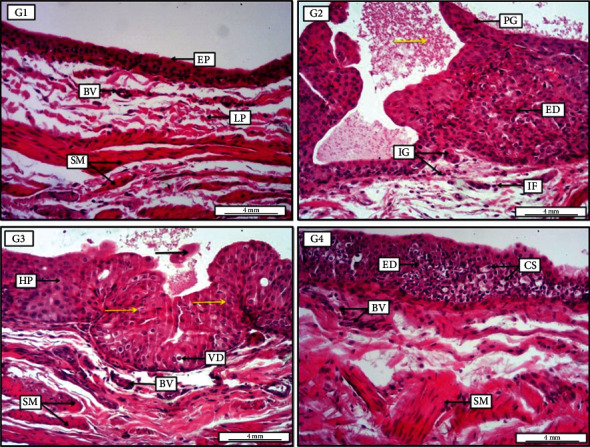
Photomicrograph of urinary bladder at week 12 from groups (G1-G4 are used for simplicity in the photomicrograph). G1, negative control group receiving only water for 12 weeks, demonstrates no prominent lesions, apparent by typical transitional epithelium (EP), with standard loose connective tissue of lamina propria (LP) which contain some blood vessels (BV), and normally oriented smooth muscle bundles (SM). G2, positive control group receiving N-butyl-N-(4-hydroxybutyl) nitrosamine (BBN) for 9 weeks, shows significant high grade of epithelial dysplasia (ED), together with papillary-like growth in the lumen (PG). The section also reveals infiltration growth toward the submucosa (IG), in addition to the present of some inflammatory cells (IF) within the lamina propria and pinkish proteinaceous materials within the lumen (yellow arrow). G3, preventive group receiving BBN for 9 weeks with thymoquinone 25 mg/kg for 10 weeks, displays low-grade epithelial hyperplasia (HP), together with significant area of urothelial dysplasia (yellow arrows); in addition, there is clear vacuolar degeneration within the dysplastic urothelial cells. Presence of pinkish sloughed epithelial cells mixed with fluid in the lumen; also, there is an evidence of smooth muscle fiber proliferation (SM), with vascular congestion (BV). G4, preventive group receiving BBN for 9 weeks with thymoquinone 50 mg/kg for 10 weeks, expresses the presence of low-grade epithelial dysplasia (ED) together with significant acute cellular swelling (CS) within the dysplastic epithelia, in addition to the presence of some newly formed blood vessels within the submucosa (BV). Moreover, the section shows mild smooth muscle fiber proliferation (SM). H&E. Scale bars: 4 mm.

**Figure 7:**
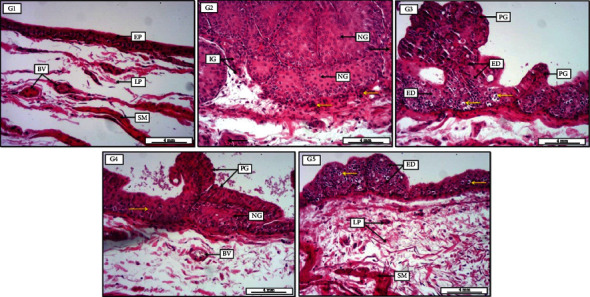
Photomicrograph of urinary bladder at week 20 from groups (G1-G5 are used for simplicity in the photomicrograph). G1, negative control group receiving only water for 20 weeks, displays no significant morphological changes, evident by normally arranged mucosal surface consist of transitional epithelium (EP), together with typically appeared lamina propria with some mild vascular congestion (BV) and smooth muscle fibers. G2, positive cancer group receiving N-butyl-N-(4-hydroxybutyl) nitrosamine (BBN) for 9 weeks, shows highly significant preneoplastic glandular-like growth (NG), manifest by large acinar-like proliferation, together with infiltration growth (IG) toward the lamina propria; moreover, the area has been infiltrated with many mononuclear inflammatory cells (yellow arrows). Additionally, there is moderate vascular congestion within the lamina propria (black arrows). G3, preventive group receiving BBN for 9 weeks with thymoquinone 25 mg/kg for 10 weeks, demonstrates significant high-grade epithelial dysplasia (ED) together with papillary-like pattern of preneoplastic growth (PG). Additionally, presence of clear cellular swelling within the lining epithelium of the bladder (yellow arrows). G4, preventive group receiving BBN for 9 weeks with thymoquinone 50 mg/kg for 10 weeks, reveals low-grade epithelial dysplasia (yellow arrow) together with moderate papillary-like growth (PG) within the dysplastic epithelia, as well as the presence of preneoplastic pattern of growth (NG). Besides, the section shows mild vascular congestion (BV). G5, preventive-treatment group receiving BBN for 9 weeks with thymoquinone 50 mg/kg for 20 weeks, displays significantly low-grade epithelial dysplasia (ED) with no evidence of preneoplastic growth, along with apparent cellular swelling within the lining epithelium (yellow arrows). In addition, the section demonstrates typically intact lamina propria (LP) with mild inflammatory cells and normally appeared smooth muscle fibers (SM). H&E. Scale bar: 4 mm.

Basically, microscopical examination of urothelial neoplastic growth was measured quantitatively in respect to some morphometric changes including hyperplasia, dysplasia, papillary, and infiltrative growth together with atypia and neoplastic transformation. The lesion scoring and grading system of bladder sections were illustrated and discussed specifically in Table 1. At week 12 of experimental point, urothelial sections in PN-50 group preventive approach treated with 50 mg/kg TQ orally for 10 weeks show significant (*P* < 0.05) alleviation in the percentage of dysplastic and preneoplastic growths in comparison to cancer control group (PC), apparent by remarkable mitigation in the number of hyperplastic and dysplastic cells, as well as papillary and infiltration preneoplastic growth (Figure 6). Additionally, animals treated with TQ 25 mg/kg (PN-25) for 10 weeks as a preventive measure also reveal significant alleviation in lesion scoring and neoplastic grading in comparison with control positive group (PC). Interestingly, at 20th week of the experimental period, animals in PN-Treatment 50 that received 50 mg/kg TQ orally for 20 weeks as a preventive and treatment measure show remarkable as well as significant amelioration in the abnormal preneoplastic growth, evident by obvious reduction in the area of hyperplasia, high-grade dysplasia, papillary-like growth along with infiltrative, and carcinoma in situ growth in the urothelial layers. These findings were more significant (*P* < 0.05) in PN-Treatment 50 compared to PN-25 and PN-50 preventive approaches, in which animals from these groups supplemented with 25 and 50 mg/kg of TQ, respectively, for only 10 weeks starting from day one (Figure 7). In conclusion, the results strongly suggest that simultaneous administration of 50 mg/kg TQ for 20 weeks has a significant effect in reduction of neoplastic and preneoplastic transformations as a preventive or prophylactic measure.

### 3.4. Serum Level of TOS and NF-*κ*B

**Figure 8:**
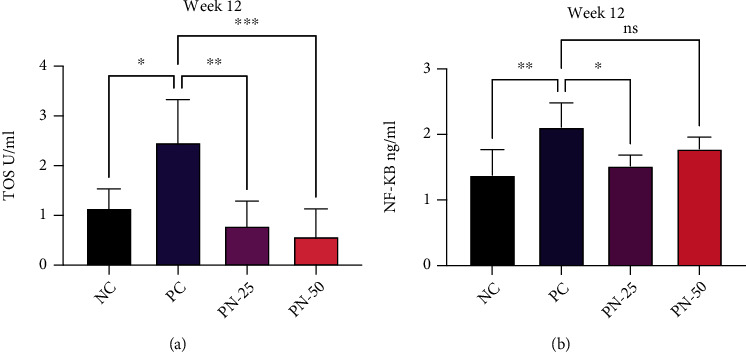
Serum level of (a) total oxidant status (TOS) in different groups. (b) Nuclear factor kappa B (NF-*κ*B) in different groups at week 12. Values are presented as mean ± SEM. ^∗^*P* value < 0.03, ^∗∗^*P* value < 0.002, and ^∗∗∗^*P* value < 0.003 are statistically significant.

At the end of week 12, oxidative stress and inflammatory status of all animals were evaluated via measurement of TOS and NF-*κ*B. In the BBN-treated group, there was a significant elevation of the serum level of TOS and NF-*κ*B when compared to the NC group (*P* < 0.05). However, in the TQ preventive groups (PN-25 and PN-50), a significant reduction in TOS and NF-*κ*B levels has been observed in comparison to the PC group, and the better outcome was noted in the PN-50 group in respect of TOS, while the serum level of NF-*κ*B decreased more in the PN-25 group (Figures 8(a) and 8(b)).

**Figure 9:**
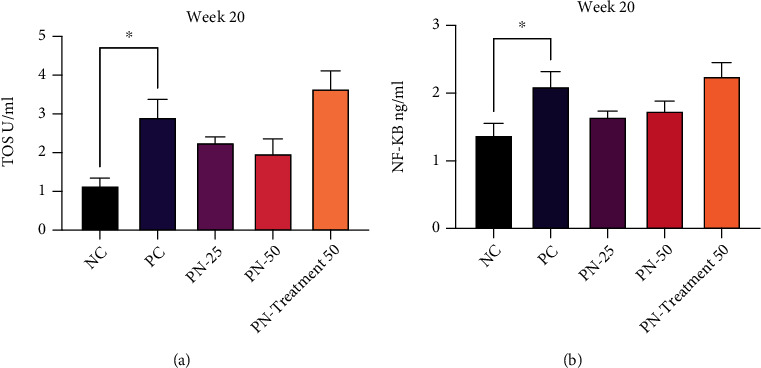
Serum level of (a) total oxidant status (TOS) in different groups at week 20. (b) Nuclear factor kappa B (NF-*κ*B) in different groups at week 20. Values are presented as mean ± SEM. ^∗^*P* value < 0.03 and ^∗∗^*P* value < 0.002 are statistically significant.

At the end of week 20, TQ preventive protocol for 10 weeks led to a decrease in serum TOS level in phase two in a nonsignificant manner. Surprisingly, TQ preventive and treatment protocol for 20 weeks resulted in a nonsignificant increase in the level of TOS in the blood. Similar results were observed in respect of NF-*κ*B biomarker (Figures 9(a) and 9(b)).

### 3.5. Serum Level of VEGF

**Figure 10:**
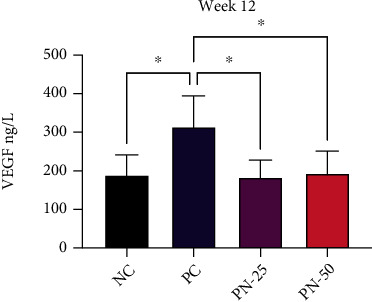
Serum level of vascular endothelial growth factor (VEGF) at week 12. Values are presented as mean ± SEM. ^∗^Significantly different between each group using one-way ANOVA followed by Tukey's multiple comparison test; *P* < 0.05 is considered as statistically significant.

In phase one and at the end of week 12, there was a significant increment in serum level of VEGF (*P* < 0.05) in the PC group, when compared with the NC group (Figure 10). In the PN-25 and PN-50 in which TQ was given as a preventive measure for 10 weeks, serum level of VEGF was significantly deceased in both doses when compared with the PC group.

**Figure 11:**
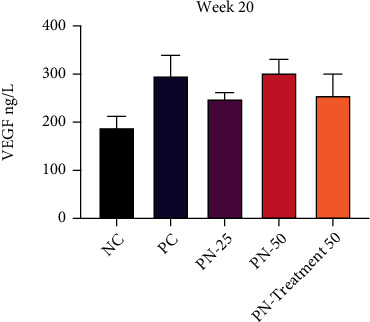
Serum level of vascular endothelial growth factor (VEGF) at week 20. Values are presented as mean ± SEM. ^∗∗^Significantly different between each group using one-way ANOVA followed by Tukey's multiple comparison test; *P* < 0.05 is considered as statistically significant.

However, in phase two and precisely at the end of week 20, serum level of VEGF was increased (*P* > 0.05) in the PC group in a nonsignificant manner, when compared with the NC group. TQ preventive trial for 10 weeks as a preventive measure in the PN-25 group and TQ preventive and treatment trial in the PN-Treatment 50 group, respectively, decreased the serum level of VEGF nonsignificantly, while it was not altered in the PN-50 group when 50 mg/kg of TQ was given for 10 weeks (Figure 11).

## 4. Discussion

Extensive experimental studies on thymoquinone (TQ) have demonstrated a broad pharmacological actions such as antioxidant [17, 18] and anti-inflammatory activity [19]. Additionally, TQ has been verified to possess anticancer activity against numerous cancers such as colorectal cancer [20], prostate cancer [21], pancreatic [22], and many others [23, 24].

In the present study, oral administration of TQ, as a preventive approach, concomitantly with the BBN carcinogen during cancer initiation exhibited a remarkable chemopreventive effect against bladder cancer. This was evidenced by macroscopical and microscopical analysis, amelioration of the oxidative stress, and inflammation that accompanied cancer induction. It is apparent that oxidative stress and inflammation could lead to cancer [25], and most contributing factors in carcinogenesis are overproduction of reactive species, inflammation, angiogenesis, and dysregulation of the balance in cellular proliferation and apoptosis. Chronic inflammation plays an important role in cancer development; therefore, overcoming the inflammatory pathways is an important mechanism of an anticancer molecule. In the present study, TQ in both studied doses and in phase one protocol was significantly alleviated the total oxidative status (TOS); however, the effect of higher dose 50 mg/kg was more significant in reduction of the serum TOS level. This finding is in tune with the several previous studies in which TQ displayed antioxidant potential against carcinogen-induced oxidative injury via various mechanism including upregulation of antioxidant and cytoprotective enzyme levels such as superoxide dismutase, catalase, glutathione reductase, and glutathione peroxidase that can combat the oxidative stress-induced tumorigenesis [26, 27]. Additionally, in another study, supplementation of TQ attenuated hepatic carcinogenesis induced by diethylnitrosamine and decreased the oxidative damage triggered by microcystin in liver tissue due to antioxidant signaling and reduced malondialdehyde, while inducing glutathione levels and superoxide dismutase, catalase, and glutathione peroxidase activities [28, 29]. Furthermore, the antioxidant activity of TQ has been shown to play a chemopreventive role in many other cancer models including colon and hepatic cancers [27, 28].

Although many studies have linked the anticancer property of TQ to its antioxidant potential, emerging evidence suggests that TQ acts as a prooxidant in cancer cells which exerts oxidative damage resulting in apoptosis; this concept supported prooxidant property of TQ as anticancer mechanism rather that antioxidant one [30, 31]. In this study, supplementation of 50 mg/kg of TQ with and after cessation of BBN for 20 weeks surprisingly resulted in elevation of oxidative status; this might explain the prooxidant capacity of TQ in amelioration of carcinogenicity in the long-term use of TQ. A large number of studies have reported the impact of the prooxidant property of TQ in its chemopreventive and chemotherapeutic actions [32, 33]. In redox cycling, through enzymatic and nonenzymatic reaction, TQ converts to prooxidant as well as antioxidant derivatives [32, 34]. With transformation of TQ into semiquinone, many reactive oxygen species (ROS) are released; these molecules resulted in oxidative stress, mitochondrial and nuclear destruction, and then subsequent cell death. It is difficult to distinguish between the prooxidant and antioxidant effects of TQ in observed anticancer response because both antioxidant and prooxidant derivatives of TQ have been reported to produce anticarcinogenic effects via various signaling pathways [8, 9].

The other interesting finding of this study was that the use of TQ in the doses of 25 mg and 50 mg/kg for 10 weeks in phase one protocol remarkably reduced the serum level of inflammatory cytokine NF-*κ*B which might be considered as one of the key mechanisms of TQ's antineoplastic actions. A recent systematic review determined TQ as a molecule that possesses anti-inflammatory and immunomodulatory activities, and it modulates inflammatory cytokines such as nuclear factor kappa B (NF-*κ*B), tumor necrosis factor-*α* (TNF-*α*), many interleukins, and certain growth factors [35]. The finding of our study is in agreement with results of the previous *in vitro* and *in vivo* study which clarified the noticeable preventive and antitumor effect of TQ on urothelial cancer through the mitigation in expression of NF-*κ*B and its regulated molecules [36]. Our finding is also being supported by several preclinical investigations on TQ which act as an anticancer molecule by hindering the NF-*κ*B signaling cascade [36–38].

However, in phase two protocol in which the follow-up of the animals was for 20 weeks, the short-term preventive approach of TQ resulted in nonsignificant alleviation of NF-*κ*B, while long-term approach in which TQ was supplemented for 20 weeks (prior and post BBN administration) resulted in elevation of this transcriptional factor. This could be explained by the presence of a complex interplay between the transcription factor NF-*κ*B signaling and oxidative stress in which increasing TOS in the present study through increasing reactive oxygen species can act as a modulator of NF-*κ*B pathway [39] and the consequent activation of this transcription protein.

As mentioned previously, the development of new blood vessels (angiogenesis) is described as the most contributing factors in carcinogenesis. It is governed by various growth factors such as fibroblast growth factors, transforming growth factor, hepatocyte growth factor, tumor necrosis factor (TNF-*α*), angiogenin, interleukin-8, and angiopoietins, and among all of these, one of the most important factors that contributes to angiogenesis is vascular endothelial growth factor (VEGF) [40]. Despite its inhibiting roles against the initiation, cell proliferation, and progression of carcinogenesis, TQ has achieved a promising hallmarks to be an alleviator of angiogenesis and metastasis processes [23, 33, 41].

In phase one of this study, TQ attenuated the serum level of VEGF in low (25 mg/kg) and high dose (50 mg/kg). However, this effect was less observed in phase two in all the studied protocols; hence, higher doses of TQ with longer duration may be needed to prevent angiogenesis process. Attenuation of this principal angiogenic growth factor was also predicted in an *in vivo* study performed by Al-Trad et al. who reported the potential prophylactic role of TQ against the development of benign prostatic hyperplasia (BPH) in six Wistar rats administering 50 mg/kg TQ orally for two weeks. The finding of that study emphasized on the TQ's ability in the amelioration of VEGF level in the treated group as well as prostate weight/body weight ratio, epithelial hyperplasia, serum interleukin 6 (IL-6) levels, and the expressions of TGF-1 [42]. Additionally, our finding is supported by the previous *in vivo* study in which TQ treatment significantly decreases angiogenesis via regulating the signal of VEGF through Akt and extracellular receptor kinase pathway [37].

As the gold standard, the histopathological analysis of urinary bladder urothelium in the present study showed a significant (*P* < 0.05) alleviation in the percentage of dysplastic and preneoplastic growths in short-term TQ preventive protocol in phase one while in long-term TQ preventive protocol in phase two, and the better outcome was noted in long-term administration of TQ for the entire experiment period. This finding is consistent with most of the biochemical results in the present study in which TQ exerted a remarkable prevention in initiation of tumors through attenuation of TOS, NF-*κ*B, and angiogenesis in phase one and suppression of tumor growth through modulation of these biomarkers in phase two.

In the present study, the restoration of the histopathological alteration of urinary bladder with TQ is consistent with the findings of the other cancer-induced studies that used TQ against carcinogenesis of colon [16], bladder [36], renal [43], and many others [41].

The effect of TQ on bodyweight has showed a nonsignificant difference in the weight gain between the studied groups in both phases and during the entire experiment; therefore, TQ has a neutral effect on this anthropometric parameter; this finding is consistent with the results of an animal study which investigated the effect of *Nigella sativa* extracts and TQ along with high-fat diet on bodyweight; the results suggested that the weight loss in the studied groups is less dependent on TQ, and it seems that other compounds present in the extracts were responsible for decreasing the bodyweight [44].

## 5. Conclusion

Thymoquinone inhibited the development of neoplastic and preneoplastic transformations; as a result, it suppressed premalignant and malignant lesion formation in a rat model of bladder cancer, which might be due to antioxidant, prooxidant, and anti-inflammatory properties of TQ. The better outcome was obtained by concurrent administration of TQ with BBN in high dose (50 mg/kg) for 20 weeks. This finding is promising in implementation of TQ alone or as adjuvant therapy for chemoprevention against the development of bladder cancer.

## Figures and Tables

**Table 1:** Histological quantitative evaluation of urothelium neoplastic changes in different time lines.

Experimental groups (*N* = 10)	Hyperplasia^#^ (mean %)^∗∗^	Dysplasia^#^ (mean %)^∗∗^	Papillary growth^∗^ (mean %)^∗∗^	Infiltrative growth^∗^ (mean %)^**^	Carcinoma in situ (Cis)^∗^ (mean %)^∗∗^	Lesion scoring (0-100%)	Lesion grading	Neoplastic grading
Week 12								
NC†	1.24%^A^	0.61%^A^	0.78%^A^	0.06%^A^	0.03%^A^	0-10%	Normal	No atypia
PC	67.34%^D^	74.29%^D^	68.33%^D^	56.83%^D^	52.82%^D^	50-75%	High grade	Cis
PN-25	49.22%^C^	50.56%^C^	47.17%^C^	44.62%^C^	39.41%^C^	25-50%	Medium grade	Dysplasia
PN-50	48.57%^C^	50.01%^C^	48.26%^C^	42.76%^C^	37.94%^C^	25-50%	Medium grade	Dysplasia

Week 20								
NC†	2.56%^A^	0.38%^A^	0.59%^A^	0.08%^A^	0.04%^A^	10-50%	Normal	No atypia
PC	75.48%^E^	81.43%^E^	77.81%^E^	84.25%^E^	92.46%^E^	75-100%	Critical grade	Invasive
PN-25	58.23%^D^	69.72%^D^	71.64%^D^	64.72%^D^	62.47%^D^	50-75%	High grade	Cis
PN-50	57.92%^D^	59.81%^D^	68.57%^D^	54.46%^D^	58.35%^D^	50-75%	High grade	Cis
PN-Treatment 50	34.87%^C^	44.72%^C^	36.28%^C^	28.54%^C^	23.67%^B^	25-50%	Medium grade	Dysplasia

Notes: ∗areas of papillary-like growth, infiltrative growth, and carcinoma in situ were estimated in (*μ*m). ^#^Hyperplasia and dysplasia were estimated in calculated counted cell number. ∗∗Each value represents mean percentage of *n* = 10. Statistical comparison among groups: mean values with different capital letters have significant differences at *P* < 0.05. ^†^NC: negative control group received (only water); PC: positive control group received N-butyl-N-(4-hydroxybutyl) nitrosamine (BBN) for 9 weeks; PN-25: preventive group received BBN for 9 weeks and thymoquinone 25 mg/kg for 10 weeks; PN-50: preventive group received BBN for 9 weeks and thymoquinone 50 mg/kg for 10 weeks; PN-Treatment 50: preventive-treatment group received BBN for 9 weeks and thymoquinone 50 mg/kg for 20 weeks.

## Data Availability

All data supporting the results of the study are available from corresponding author upon request.
